# Metabolite Profiling in *Arabidopsis* *thaliana* with Moderately Impaired Photorespiration Reveals Novel Metabolic Links and Compensatory Mechanisms of Photorespiration

**DOI:** 10.3390/metabo11060391

**Published:** 2021-06-15

**Authors:** Stefan Timm, Adriano Nunes-Nesi, Alexandra Florian, Marion Eisenhut, Katja Morgenthal, Markus Wirtz, Rüdiger Hell, Wolfram Weckwerth, Martin Hagemann, Alisdair R. Fernie, Hermann Bauwe

**Affiliations:** 1 Plant Physiology Department, University of Rostock, Albert-Einstein-Straße 3, 18059 Rostock, Germany; martin.hagemann@uni-rostock.de; 2 Max Planck Institute of Molecular Plant Physiology, Am Mühlenberg 1, D-14476 Golm, Germany; nunesnesi@ufv.br (A.N.-N.); florian.alexandra@web.de (A.F.); morgenthal@mpimp-golm.mpg.de (K.M.); wolfram.weckwerth@univie.ac.at (W.W.); fernie@mpimp-golm.mpg.de (A.R.F.); 3 Departamento de Biologia Vegetal, Universidade Federal de Viçosa, Viçosa 36570-900, Minas Gerais, Brazil; 4 Institute of Plant Biochemistry, Cluster of Excellence on Plant Science (CEPLAS), Heinrich Heine University Düsseldorf, Universitätsstrasse 1, 40225 Düsseldorf, Germany; m.eisenhut@uni-duesseldorf.de; 5 Centre for Organismal Studies, University of Heidelberg, Im Neuenheimer Feld 360, D-69120 Heidelberg, Germany; markus.wirtz@cos.uni-heidelberg.de (M.W.); ruediger.hell@cos.uni-heidelberg.de (R.H.); 6 Department of Molecular Systems Biology, University of Vienna, Althanstrasse 14, 1090 Vienna, Austria

**Keywords:** *Arabidopsis*, photorespiration, hydroxypyruvate reductase, metabolomics, isotope labeling, metabolic acclimation, plant development, photoperiodic acclimation

## Abstract

Photorespiration is an integral component of plant primary metabolism. Accordingly, it has been often observed that impairing the photorespiratory flux negatively impacts other cellular processes. In this study, the metabolic acclimation of the *Arabidopsis* *thaliana* wild type was compared with the hydroxypyruvate reductase 1 (HPR1; *hpr1*) mutant, displaying only a moderately reduced photorespiratory flux. Plants were analyzed during development and under varying photoperiods with a combination of non-targeted and targeted metabolome analysis, as well as ^13^C- and ^14^C-labeling approaches. The results showed that *HPR1* deficiency is more critical for photorespiration during the vegetative compared to the regenerative growth phase. A shorter photoperiod seems to slowdown the photorespiratory metabolite conversion mostly at the glycerate kinase and glycine decarboxylase steps compared to long days. It is demonstrated that even a moderate impairment of photorespiration severely reduces the leaf-carbohydrate status and impacts on sulfur metabolism. Isotope labeling approaches revealed an increased CO_2_ release from *hpr1* leaves, most likely occurring from enhanced non-enzymatic 3-hydroxypyruvate decarboxylation and a higher flux from serine towards ethanolamine through serine decarboxylase. Collectively, the study provides evidence that the moderate *hpr1* mutant is an excellent tool to unravel the underlying mechanisms governing the regulation of metabolic linkages of photorespiration with plant primary metabolism.

## 1. Introduction

Plants are exposed to a frequently changing environment throughout all stages of development. Among others, this especially includes sudden alterations in the prevailing temperature, water and nutrient availability, the CO_2_/O_2_ ratio and light availability. Therefore, dynamic regulation of metabolism to either long- or short-term environmental changes is required to sustain metabolic robustness for efficient photosynthesis and growth [1,2,3]. In this regard, photorespiration emerged as one of the key determinants for an optimal acclimation capacity towards a multitude of environmental factors in oxygenic phototrophs [4,5,6,7]. This is because the pathway represents the second highest carbon flux during illumination and is orchestrated in four different subcellular compartments (chloroplasts, peroxisomes, mitochondria and the cytoplasm) and as such eventually impacts on a multitude of other processes [8,9,10].

The term photorespiration describes the light-induced CO_2_ release and O_2_ uptake in C3 plants. The photorespiratory metabolism is mainly required to remove 2-phosphoglycolate (2PG), the major side product of ribulose-1,5-bisphosphate (RuBP) carboxylase/oxygenase (RubisCO) in the O_2_-rich atmosphere. This process is essential because 2PG severely inhibits key enzymes involved in carbon assimilation and utilization pathways, such as the Calvin-Benson cycle, glycolysis and starch metabolism [11,12,13,14]. Accordingly, acceleration of the photorespiratory flux, i.e., faster 2PG degradation, stimulates the operation of the afore-mentioned pathways, which ultimately culminates in improved photosynthesis, growth and abiotic stress tolerance [13,15,16,17]. However, the significance of photorespiratory metabolism is not restricted to 2PG removal alone. A broad line of evidence exists for the contribution of photorespiration to the operation of other metabolic and physiological processes. To date, well-established intercepts are reported for C1 carbon metabolism [18] and nitrogen assimilation [19,20], while initial evidence was recently obtained that optimal photorespiration is also needed for the assimilation of sulfur [21]. Furthermore, it was demonstrated that photorespiration strongly impacts on the overall cellular redox-homeostasis [22], the abiotic stress tolerance [5,6], the stomatal movements [7], and was suggested to play a role in photoprotection [4]. Hence, it is reasonable to conclude that photorespiration makes a major contribution to the metabolic robustness of the plant during different stages of development.

Interestingly, comprehensive studies on different photorespiratory mutants during high to low CO_2_ transition [7,23] and experiments with wild-type plants exposed to environmental conditions [24,25] that promote photorespiration (i.e., RuBP oxygenation) suggested further links of the pathway with other metabolic branches. For instance, clear metabolic alterations were frequently observed in the tricarboxylic acid (TCA) cycle, amino acid metabolism or the urea cycle in response to impaired photorespiration [26,27,28]. While such responses were observed in independent studies, the regulatory mechanisms governing such metabolic linkages are still largely unknown and require further attention [29,30]. Enlarging our current understanding of the underlying regulatory mechanisms is thus mandatory to enhance overall metabolic robustness of plant metabolism towards future climate scenarios [31,32]. For such purposes, mutants showing only moderate photorespiratory phenotypes are seemingly the most valuable tools, because most pathway deletion mutants cannot survive under ambient conditions and show many pleiotropic responses [7,23].

In this study, the metabolic acclimation of *Arabidopsis thaliana* (Arabidopsis) with normal and moderately impaired photorespiration was analyzed. For this purpose, relative and absolute metabolite quantification were combined with isotope labeling approaches. Air-grown Arabidopsis wild-type plants (Col-0) were compared with the *hpr1* single mutant (deficient in hydroxypyruvate (3HP) reductase 1; HPR1—At1g68010) at different developmental stages and photoperiods. Metabolite profiling during development reveals that impairing the photorespiratory flux is more critical during vegetative growth compared to the reproductive phase and that a shorter photoperiod seems to slowdown the photorespiratory metabolite conversion compared to long days, especially due to altered regulation of glycerate 3-kinase (GLYK) and the glycine decarboxylase (GDC) activity. Despite moderately impaired photorespiration and growth of *hpr1* in air [33,34], we demonstrate that the mutant displays severe alterations in the leaf-carbohydrate status, impacting on carbohydrate derivates related to cell wall biosynthesis, and a significantly higher fraction of CO_2_ release from illuminated leaves. The obtained results are discussed mainly in the context of novel interactions of photorespiration, such as those with cell wall and ethanolamine (ETA) biosynthesis, as well as additional decarboxylation reactions in response to impaired photorespiration, mainly non-enzymatic decarboxylation of 3HP. The most critical photorespiratory steps for acclimation towards altered photoperiods were also determined.

## 2. Results

To assess the impact of moderately impaired photorespiration on the metabolic acclimation capacity of Arabidopsis, especially during developmental and photoperiodic alterations, three sets of experiments were designed. First, targeted absolute metabolite quantification combined with [U-^13^C]-glucose and [U-^14^C]-glycine labeling approaches were carried out to obtain a higher resolution of the key metabolic changes in *hpr1* compared to the wild type (Col-0) grown in normal air. Second, metabolic profiles of the wild type with the *hpr1* mutant during different developmental stages were compared. Third, diurnal metabolic cycles of selected photorespiratory intermediates of the wild type and the *hpr1* mutant grown in air under two different photoperiods were analyzed.

### 2.1. Absolute Metabolite Quantification and ^13^C-Glucose Feeding Suggests Enhanced Non-Enzymatic 3HP Decarboxylation and Increased Flux towards Ethanolamine in hpr1

**Figure 1:**
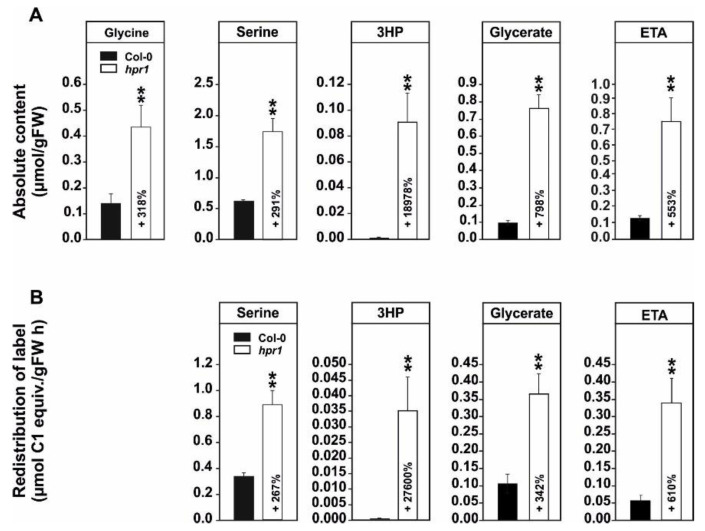
Absolute amounts and carbon isotope redistribution in photorespiratory intermediates and ethanolamine. Shown are (**A**) absolute amounts of selected photorespiratory intermediates and ethanolamine (ETA) and (**B**) redistribution of ^13^C in selected photorespiratory intermediates (serine (flux from glycine), 3HP (flux from serine) and glycerate (flux from 3HP)) and ETA (flux from serine) following [U-^13^C]-glucose labeling in the wild type and the *hpr1* mutant. Plants were grown under controlled environmental conditions (390 ppm CO_2_, ~120 µmol m^−2^ light intensity, 20/18 °C day/night temperature, ~70% relative humidity) for 5 weeks (12/12 h day/night cycle). At this time point, leaf-discs were obtained for absolute metabolite quantification and carbon isotope labeling. Shown are means ± SD of five biological replicates (5 leaf-discs per plant) and asterisks show significant changes compared with the wild type according to Student’s *t*-test (** *p* < 0.01).

Compared to most other photorespiration mutants, previous metabolome studies on the *hpr1* mutant revealed only very few, but specific, metabolic changes, demonstrating its value to specifically analyze metabolic interactions of photorespiration [23,33,35]. However, in all these studies, metabolites were determined only as relative changes. In order to gain a higher resolution of the metabolic alterations in *hpr1*, especially of those linked to the photorespiratory pathway, an absolute metabolite quantification approach using plants grown in normal air was employed. As shown in Figure 1A, *hpr1* showed significant increases in the absolute amounts of photorespiratory intermediates including glycine (3.2-fold), serine (2.9-fold), and glycerate (8.0-fold). Expectedly, the strongest relative increase was seen in 3HP (190-fold). Given that some alterations in the ethanolamine (ETA) content was observed during previous studies [23,33], this intermediate was also in focus. Interestingly, an approximately 5.5-fold higher absolute amount of ETA was determined in *hpr1*.

Increased steady-state amounts can originate from increased synthesis or decreased breakdown, the latter is clearly the case for 3HP in the *hpr1* mutant. To directly gain insights in the turnover of the mentioned intermediates, the incorporation of ^13^C in those representatives was measured following [U-^13^C]-glucose feeding. The relative ^13^C incorporation into serine (2.7-fold; flux from glycine), glycerate (3.4-fold; flux from 3HP) and ETA (6.1 fold; flux from serine) was significantly enhanced in the mutant *hpr1* compared to the wild type. Interestingly, all flux enhancements were in a comparable range as the increases in the absolute values. Accordingly, the increase in steady-state levels of these intermediates is well supported by an increased ^13^C incorporation into these pools. Exceptionally, a significantly higher proportion of ^13^C incorporation into 3HP (276-fold; flux from serine) in *hpr1* was measured compared to the increase in its steady-state level (Figure 1B). In the case of 3HP, the labeling increase was almost 60% higher than expected from its accumulation, which indicates an increased breakdown of 3HP in the mutant independent from the HPR1 reaction. This statement agrees well with a decrease (~57%) in ^13^C labeling in glycerate if compared to its absolute steady-state amount (Figure 1A,B).

### 2.2. ^14^C-Glycine Labeling Reveals a Strong Reduction of the Leaf Carbohydrate and Organic Acid Status and an Increased CO_2_ Release from hpr1 Leaves

**Figure 2:**
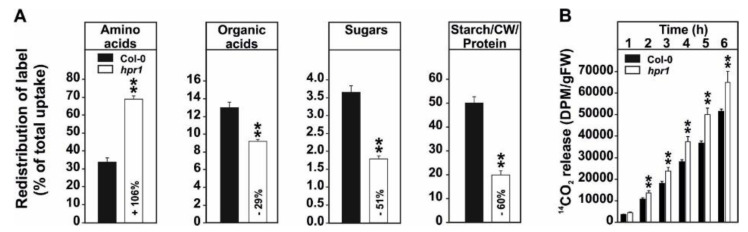
Carbon isotope redistribution following ^14^C-glycine-labeling. Shown is (**A**) the redistribution of ^14^C in four major fractions of cellular compounds and (**B**) the ^14^CO_2_ evolved from leaf-discs harvested from the wild type in comparison with the *hpr1* mutant. Plants were grown under controlled environmental conditions (390 ppm CO_2_, ~120 µmol m^−2^ s^−1^ light intensity, 20/18 °C day/night temperature, ~70% relative humidity) for 5 weeks (12/12 h day/night cycle). At this time point, leaf-discs were fed with [U-^14^C]-glycine for 6 h and then harvested for subsequent analysis. The ^14^CO_2_ evolved was captured (in hourly intervals) in a KOH trap and the amount of radiolabel released was subsequently quantified. Shown are means ± SD of five biological replicates (5 leaf-discs per plant) and asterisks show significant changes compared with the wild type according to Student’s *t*-test (** *p* < 0.01).

The next aim was to classify the metabolic alterations in the *hpr1* mutant through carbon isotope fractionation following [U-^14^C]-glycine labeling. For this purpose, plants grown for 5 weeks under photorespiratory conditions in normal air with a 12/12 h day/night cycle were used. In general, *hpr1* showed a significantly increased labeling in the fraction of free amino acids (+106%), while organic acids (–29%), soluble sugars (–51%), and the high molecular mass fraction including starch, the cell wall and proteins (–60%) were less labeled compared to the wild type (Figure 2A). Interestingly, *hpr1* displayed an increased CO_2_ release (~29% on average) from the leaves compared to the wild type (Figure 2B).

### 2.3. The Elevated Serine Content in hpr1 Enhances Glutathione Biosynthesis

**Figure 3:**
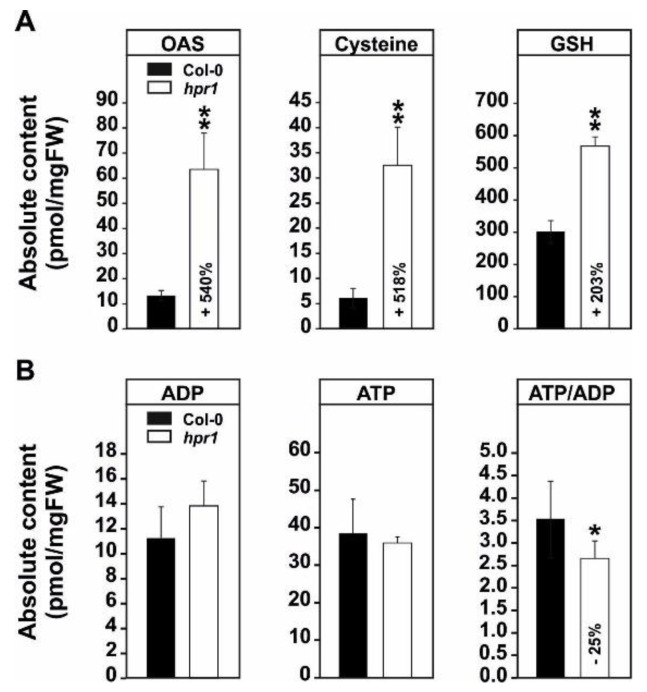
Absolute metabolite contents of selected intermediates related to serine and energy metabolism. Shown are (**A**) absolute amounts of *O*-acetylserine (OAS), cysteine and glutathione (GSH; sum parameter of GSH + GSSG) and (**B**) energy equivalents (adenosine diphosphate [ADP], adenosine triphosphate [ATP]) form air grown wild-type and *hpr1* mutant plants. Shown are means ± SD (five biological replicates) and asterisks show significant changes compared with the wild type according to Student’s *t*-test (* *p* < 0.05; ** *p* < 0.01).

In addition to the expected impact of *HPR1* deficiency on photorespiratory serine and 3HP accumulation, clear indications were observed that other pathways also respond to elevation of these intermediates, particularly serine. As shown in Figure 3A, *hpr1* accumulated *O*-acetylserine (OAS, +540%), the precursor of cysteine biosynthesis, as well as cysteine (+518%) itself. Additionally, an increase was found in glutathione (GSH; sum parameter of GSH + GSSG, +203%) in *hpr1* compared to wild type (Figure 3A). Despite these changes, only minor changes in the amounts of adenosine phosphates were detected. In detail, a slight, but non-significant, increase in ADP was seen in *hpr1*, while ATP was mostly unaltered. However, the ATP/ADP ratio was significantly reduced (~25%) in the *hpr1* mutant compared to the wild type (Figure 3B).

### 2.4. HPR1 Deficiency Increasingly Impairs Photorespiration during Vegetative Growth

**Figure 4:**
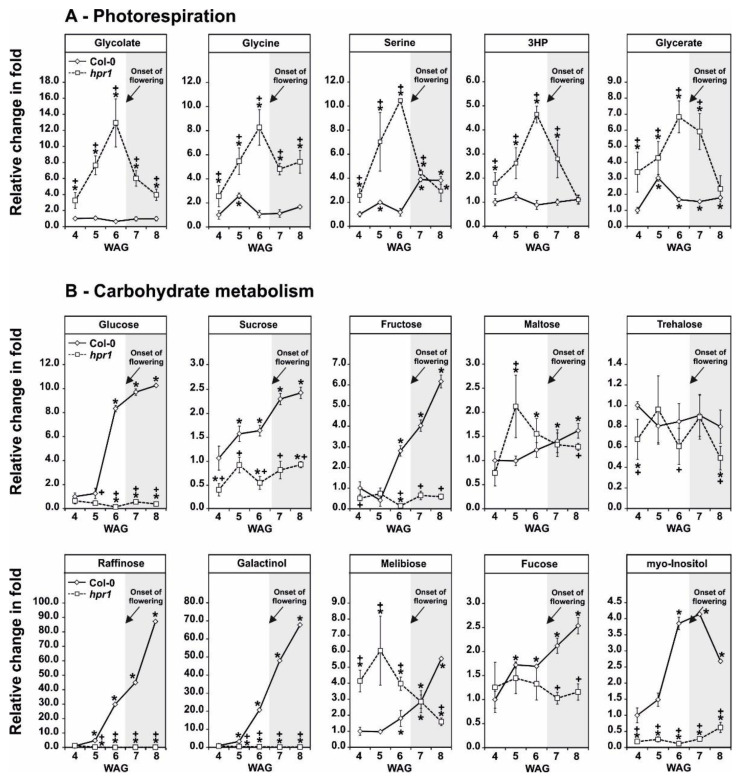
Metabolic acclimation patterns of photorespiration and carbohydrates during development. Relative metabolite abundances of selected intermediates of (**A**) photorespiration and (**B**) carbohydrate metabolism in Arabidopsis wild-type and *hpr1* mutant plants during development (3–7 weeks after germination (WAG)). Plants were grown under controlled environmental conditions (390 ppm CO_2_, ~120 µmol m^-2^ s^-1^ light intensity, 20/18 °C day/night temperature, ~70% relative humidity) in a balanced photoperiod (12/12 h day/night cycle). Mutant-to-wild-type ratios ± SD (five biological replicates) are shown (relative amounts, arbitrary units mg^−1^ fresh weight), with the mean wild-type value at 3 WAG arbitrarily set to 1 (wild type, solid line; *hpr1*, dashed line). The white shaded part of the graph represents the vegetative phase, while grey shading the reproductive phase. Asterisks show significant changes compared to the wildtype at 3 WAG and plusses (* *p* < 0.05) between the wild type and the *hpr1* mutant at the respective timepoint according to Student’s *t*-test (* *p* < 0.05).

Photorespiration is essential to activate photosynthesis already during postembryonic growth after the transition from night to day in normal air [36,37]. Hence, it seems reasonable that the pathway needs to operate efficiently in leaves throughout all stages of plant development. To analyze this from the metabolic point of view, the wild type and the *hpr1* mutant were grown in air and leaf-material harvested for subsequent metabolome analysis at five selected stages (3 to 7 weeks after germination; WAG). While material of the first three time points (3–5 WAG) is characteristic for vegetative growth, the latter two (6–7 WAG) are representative for the reproductive phase. In the wild type, five diagnostic intermediates of photorespiration (glycolate, glycine, serine, 3HP and glycerate) did not show many variations during development, except for a stable increase in serine after week 5 (Figure 4A). In contrast, the mild reduction in the photorespiratory flux in *hpr1* [34] caused a characteristic over-accumulation pattern of these photorespiratory intermediates. All pathway intermediates increased in their abundances during the vegetative phase, reaching highest levels after 5 weeks, but subsequently declined after bolting (between week 5 and 6), which induces the transition to the reproductive phase. However, glycolate and glycine remained elevated (3–5-fold) after 6–7 weeks compared to the wild type (Figure 4A).

### 2.5. Impaired Photorespiration Lowers the Accumulation of Sugars

**Figure A1:**
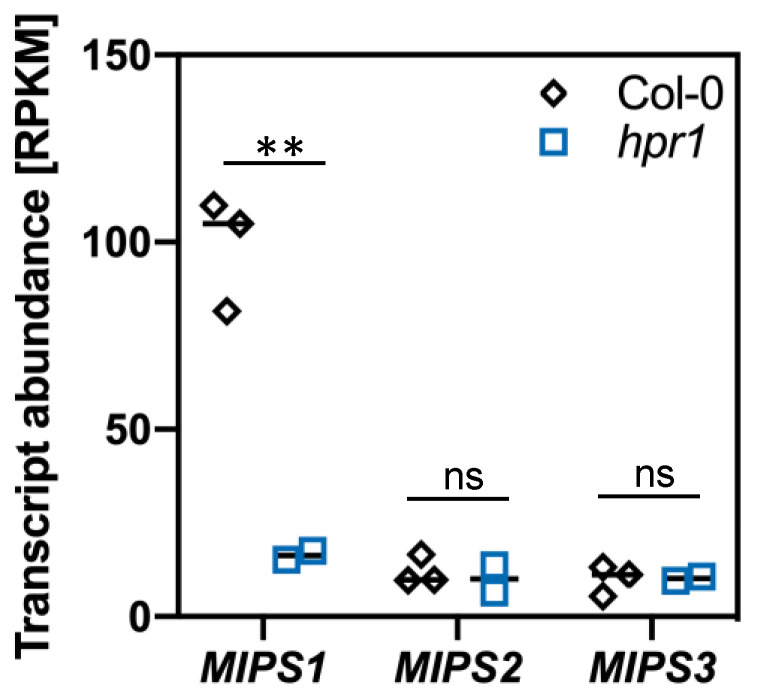
Transcript abundance of *MIPS1-3* genes in the wild type and *hpr1* 8 h after transition from elevated (1% CO_2_ in air) to normal ambient (0.038% CO_2_ in air) CO_2_ conditions. Transcript abundances are given as individual values of each biological replicate and mean of RPKM (reads per kilobase of transcript per million reads mapped). Significance was tested with the two-tailed Student’s *t*-test. ** *p* < 0.01; ns, not significant. Data was extracted from a previous study [7].

Reduction of the photorespiratory flux feeds back to the operation of photosynthesis, especially the operation of the Calvin–Benson cycle and carbon export thereof [13,38,39]. Therefore, the accumulation of soluble sugars and higher mass carbohydrates were followed. Except for trehalose, the wild type increasingly accumulated sugars including glucose, sucrose, fructose and maltose during the course of our experiment, reaching highest levels in the reproductive growth phase. A similar pattern was seen also for raffinose and selected carbohydrates and carbohydrate derivates related to cell wall metabolism including galactinol, melibiose, fucose and *myo*-inositol (Figure 4B, lower panel). In *hpr1*, glucose, sucrose and fructose were generally less abundant and present at similar amounts over the analyzed time points. Maltose differed a bit from that pattern, as it appeared slightly elevated in the early phase of the experiment but tended to decrease in the late stages. Again, no consistent change and pattern was seen in the amounts of trehalose in *hpr1* (Figure 4B, upper panel). However, HPR1 deficiency caused a strong and consistent reduction in raffinose, galactinol and *myo*-inositol over all analyzed time points. Fucose was present at similar amounts during the first two weeks, but also significantly reduced at the latter 3 time points. Melibiose showed a biphasic accumulation pattern, as it was present at higher amounts during the first three weeks but declined after transition to the reproductive growth phase (Figure 4B. lower panel). Collectively, these results demonstrate that reduction in the photorespiratory flux in *hpr1* impacts on high molecular mass carbohydrate accumulation and, possibly, cell wall biosynthesis, due to lowered substrate availability. In addition to the metabolic changes observed in course of the current study, previous characterization of *hpr1* via mRNA sequencing supports this hypothesis. The transcript abundance of the enzyme *myo*-inositol 1-phosphate synthase 1 (MIPS1, [40]), the major *myo*-inositol biosynthesizing enzyme, was strongly reduced in *hpr1* upon transition to ambient CO_2_ conditions (Figure A1; [7]).

### 2.6. HPR1 Deficiency Impacts on the TCA Cycle and Amino Acid Metabolism during Development

**Figure 5:**
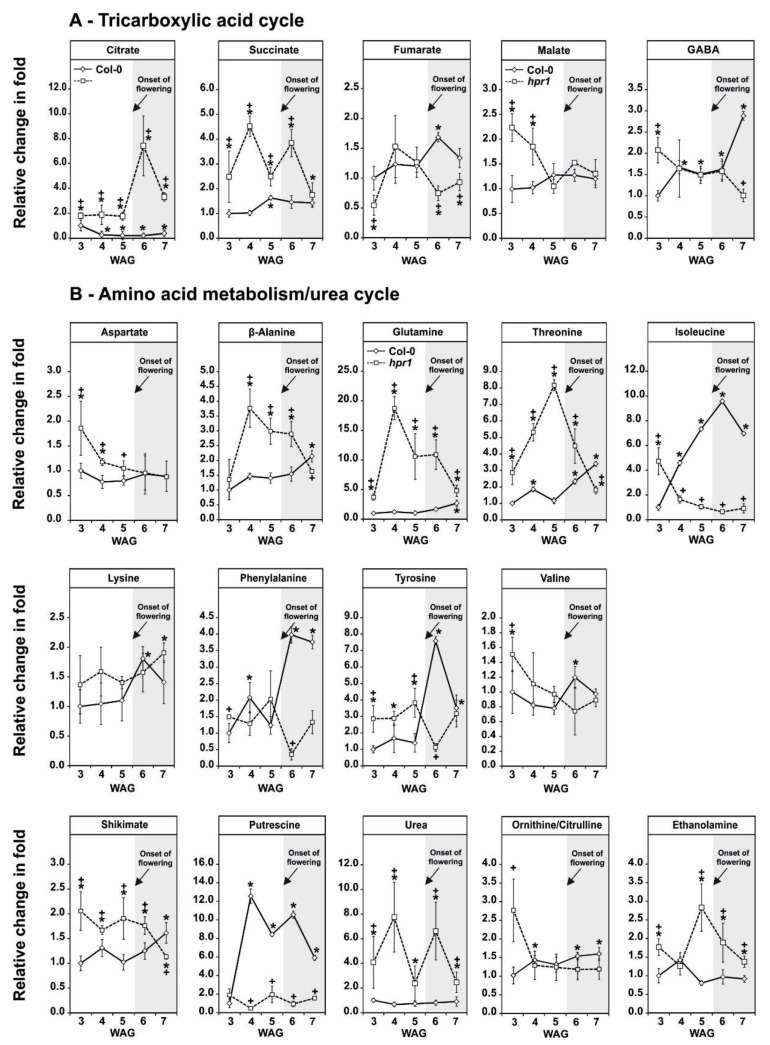
Metabolic acclimation patterns of the TCA cycle and amino acid metabolism during development. Metabolite abundances of selected intermediates of (**A**) the TCA-cycle and (**B**) amino acid metabolism and the urea cycle in Arabidopsis wild-type and *hpr1* mutant plants during development (3–7 weeks after germination [WAG]). Plants were grown under controlled environmental conditions (390 ppm CO_2_, ~120 µmol m^−2^ s^−1^ light intensity, 20/18°C day/night temperature, ~70% relative humidity) in a balanced photoperiod (12/12 h day/night cycle). Mutant-to-wild-type ratios ± SD (five biological replicates) are shown (relative amounts, arbitrary units mg^−1^ fresh weight), with the mean wild-type value at 3 WAG arbitrarily set to 1 (wild type, solid line; *hpr1*, dashed line). The white shaded part of the graph represents the vegetative phase, while grey shading the reproductive phase. Asterisks show significant changes compared with the wild-type time point, and plus signs (* *p* < 0.05) show them compared with the wild type at 3 WAG according to Student’s *t*-test (* *p* < 0.05).

An impact on the operation of the TCA cycle was previously observed in photorespiratory mutants during CO_2_ transition [7,23]. Therefore, five selected representatives of, or associated to, the pathway were profiled during development. Minor fluctuations (~1.5-fold) in citrate, succinate, fumarate and malate were seen in the wild type. Only GABA increased during the experiment, with highest levels (~2.5-fold) 7 WAG (Figure 5A). Deletion of *HPR1*, however, impacted those accumulation patterns. Citrate and succinate were generally higher in the *hpr1* mutant over time, while minor fluctuations were seen in fumarate (decreased at week 6 and 7) and malate (increased in weeks 3 and 4). The accumulation of GABA was inverse of that in the wild type, which was elevated at week 3 and gradually decreasing until week 7 (Figure 5A).

**Figure 6:**
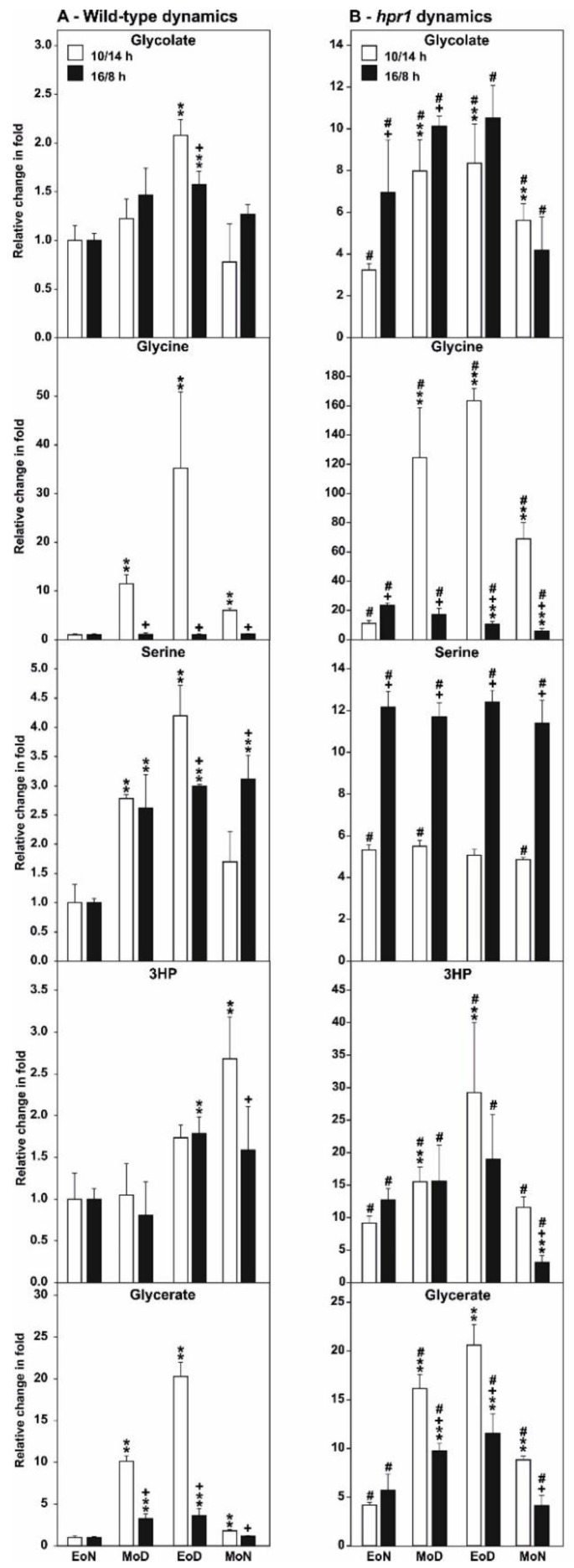
Selected intermediates of photorespiration of plants grown under two different photoperiods. Shown are relative amounts of selected photorespiratory intermediates form air grown wild-type and *hpr1* mutant plants in two photoperiods (SD—10/14 h day/night cycle, and LD—16/8 h day/night cycle) with otherwise equal conditions. Plants were grown to growth stage 5.1 [41] and metabolites determined at four diagnostic timepoints during the day/night cycle (end of night (EoN), mid of day (MoD), end of day (EoD) and mid of night (MoN)). Shown are means ± SD (five biological replicates) with the wildtype EoN time point was arbitrary set to 1. Asterisks show significant changes compared with the wild-type EoN timepoint, plusses indicate significant changes between SD and LD and rhombus indicate significant changes at the respective timepoint between the wild type and *hpr1* according to Student’s *t*-test (** *p* < 0.01). The figure was redrawn, using data that were in part previously published [35], with permission (www.plantphysiol.org).

Among the analyzed amino acids, aspartate and valine showed almost no changes over the entire period of the experiment in the wild type. However, many others continuously increase over time (glutamine, threonine, alanine, isoleucine, lysine, phenylalanine and tyrosine) with highest values in the reproductive growth phase (Figure 5B). A similar pattern was seen for the aromatic amino acid biosynthesis precursor shikimate or the amino acid breakdown product putrescine, while representatives of the urea cycle (urea, ornithine/citrulline) were largely invariant. ETA, a major integral part of important phospholipids, also showed only minor fluctuations in the wild type (Figure 5B). The slowdown in the photorespiratory flux in *hpr1* altered the wild-type patterns of specific metabolites. For example, alanine, glutamine, threonine and ETA showed an accumulation pattern comparable to typical photorespiratory intermediates in *hpr1* (Figure 4A, Figure 5B). In contrast, isoleucine, phenylalanine, tyrosine, shikimate, putrescine and ornithine/citrulline showed inverse accumulation pattern as in the wild type, while the amount of urea was elevated at all of the analyzed time points in *hpr1*. Small, but significant changes between the control and the mutant were, however, seen with aspartate, lysine and valine (Figure 6B).

### 2.7. Short Day Growth seems to Slowdown the Photorespiratory Flux Mainly at the GDC and GLYK Steps

Since daylength eventually impacts on photorespiration and overall metabolism [42,43], metabolite accumulation patterns in response to alterations of the photoperiod were examined. For this reason, the wild type and *hpr1* were grown in normal air with two different day/night-cycles (short day [SD], 10/14 h day/night cycle and long day [LD], 16/8 h day/night cycle) and samples taken at four diagnostic time points (end of night (EoN); mid of day (MoD); end of day (EoD) and mid of night (MoN), respectively) were analyzed.

Among the five representative photorespiratory intermediates (glycolate, glycine, serine, 3HP, and glycerate), specific differences in the diurnal accumulation pattern in the wild type between the two photoperiods were observed. In general, glycolate, serine and 3HP showed comparably minor fluctuations (~2–4-fold compared to EoN) during the day/night cycles, which were also very similar in both photoperiods. In detail, glycolate and serine increased in response to illumination with virtually highest amounts at EoD (Figure 6A). 3HP fluctuations were a bit less consistent given that almost equal amounts were seen at EoN and MoD, while some accumulation is visible at EoD and MoN. Apart from that, glycine and glycerate showed a clear diurnal pattern with highest increases (between 25–35-fold increase during illumination) among the photorespiratory intermediates. Interestingly, the accumulation of both intermediates was significantly pronounced in SD compared to LD (Figure 6A).

In *hpr1*, these rhythms were even stronger. Glycolate fluctuations were comparable to the wild type, with similar patterns in both photoperiods, but the contents were elevated to about 8–10-fold during illumination followed by a decline at MoN (Figure 6B). A comparable pattern is also visible for 3HP in *hpr1* in SD and LD, while the increases are distinctly higher during illumination (up to ~20–25-fold increase at EoD) as excepted due to the lack of HPR1. As described for the wild type, glycine and glycerate showed a clear diurnal rhythm in both photoperiods, again with the strongest amplitudes in SD. Most distinctly, glycine showed the highest increases in SD (up to 160-fold) at MoD and EoD. Comparable to a previous study [35], *hpr1* has almost equal amounts of serine at all timepoints during the day/night cycle in both photoperiods but its elevation was double as high in LD (~12-fold) compared to SD (~6-fold) (Figure 6B).

## 3. Discussion

The aim of this study was to gain insights into the metabolic acclimation of Arabidopsis with moderately impaired photorespiration, i.e., in a mutant deficient in peroxisomal HPR1 (*hpr1*). The *hpr1* mutant was in focus given that it is able to thrive in normal air, allowing us to particularly study the long-term metabolic acclimation without the necessity of short-term CO_2_-transitions used for most other pathway mutants before e.g., [7,23,44]. For this purpose, different metabolomics approaches during different developmental stages and photoperiods were employed. The main aim was to identify yet unknown metabolic links of photorespiration and potential compensatory mechanisms that occur in response to an impaired photorespiratory flux.

### 3.1. HPR1 Deficiency Causes Enhanced Leaf CO_2_ Release by Both, Increased Non-Enzymatic 3HP Decarboxylation and Higher Flux from Serine to ETA

First, a higher resolution on the metabolic alterations within the photorespiratory pathway, especially of that close to the HPR1 reaction through a combination of absolute metabolite quantification as well as ^13^C- and ^14^C-labeling approaches was obtained. This was done to eventually distinguish between changes in the steady-state contents and the flux through the pathway. In good agreement with previous studies [23,35], *hpr1* accumulates most photorespiratory intermediates in the range of ~3–8-fold (Figure 1A). Compared to many other pathway mutants, these alterations are relatively moderate (7, 23). This is best explained due to the operation of the cytoplasmic HPR2 bypass, compensating the vast amount of the pathway flux [34,35]. However, absolute metabolite quantification reveals a strong overaccumulation of 3HP, the HPR substrate, which was in the similar range as for other photorespiration mutants including that of glycerate 3-kinase (*glyk1*), serine hydroxymethyltransferase 1 (*shm1*), and 2-phosphoglycolate phosphatase 1 (*pglp1*) [7,13,45]. Relatively moderate metabolite changes were supported by the analysis of the redistribution of ^13^C in the same photorespiratory intermediates (glycine, serine and glycerate) following ^13^C-glucose labeling (Figure 1B). While accumulation of glycine and serine is likely caused by negative backlog on the pathway flux due to the impaired HPR reaction, accumulation in glycerate is not directly intuitive. Currently, the best explanation would be glycerate accumulation in the cytosol, resulting from enhanced 3HP reduction through HPR2. Given its reintegration into the chloroplasts by the plastidal glycolate/glycerate transporter (PLGG1) is in counter exchange with glycolate [46], the perturbed photorespiratory flux in *hpr1* might cause an imbalance in the operation of PLGG1. Notably, a significantly higher fraction of ^13^C incorporation into 3HP was observed in comparison to the change seen in the steady-state amounts in *hpr1*. This result can serve as indication for a higher 3HP turnover independent of HPR1. As a likely explanation for the observed effect, an increased non-enzymatic decarboxylation of 3HP can be anticipated. This assumption agrees well with previous studies on isolated peroxisomes [47] and the elevated CO_2_ release from *hpr1* leaves observed during this study (Figure 2B). Another mechanism by which an increased CO_2_ release from *hpr1* mutant leaves can be partially explained is the glucose-6-phosphate shunt as recently suggested [48,49]. Given the cytosolic HPR2 bypass is NADPH dependent, an upregulation in this pathway is a likely scenario in *hpr1* in order to provide more reductant for an elevated flux through the cytoplasm. However, and based on the data presented here, a partially redirected photorespiratory flux from serine could also serve as a likely additional source contributing to the elevated CO_2_ liberation from *hpr1*. Serine decarboxylase (SDC), a pyridoxal phosphate dependent enzyme, was suggested to be the major source of ethanolamine (ETA) used for choline biosynthesis in plants [50,51]. Since an increase in the steady-state ETA content and an elevated ^13^C incorporation into this intermediate in *hpr1* was observed (Figure 1A,B), it is tempting to speculate that a fraction of the photorespiratory serine leaks from the mitochondria into the cytoplasm where SDC resides [52]. This potential compensatory mechanism could also serve as explanation why *hpr1* maintains constantly equal serine levels during day/night cycles in different photoperiods (Figure 6B). Keeping the serine level under tight control is key because it negatively affects the transcription and translation of genes associated with photorespiration, i.e., the induction in response to onset of illumination [35].

### 3.2. Even a Moderate Impairment in the Photorespiratory Flux Reduces the Leaf-Carbohydrate Status including a Reduction in Metabolites Related to Cell Wall Biosynthesis

**Figure A2:**
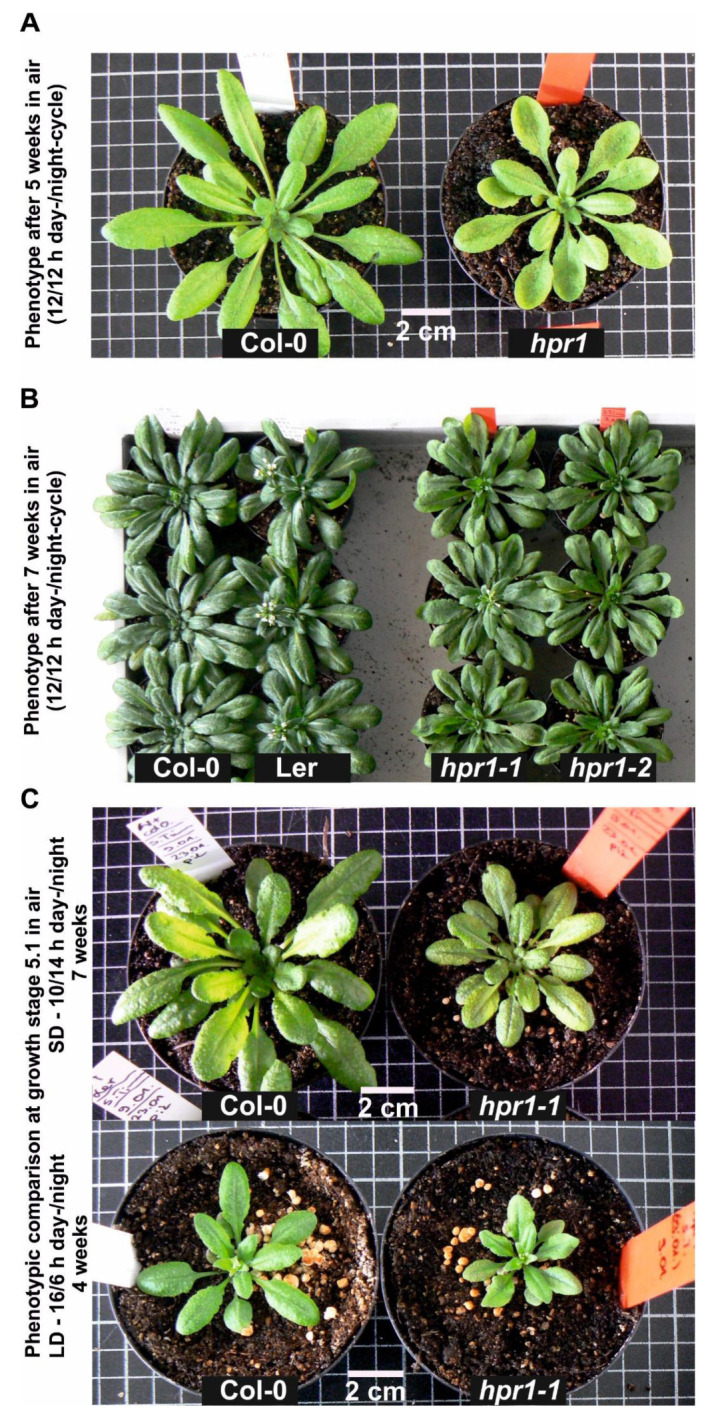
Phenotype of wild-type and *hpr1* mutant plants at different growth stages and conditions. (**A**) The wildtype in comparison to *hpr1-1* grown in normal air (390 ppm CO_2_) for 5 weeks in a 12/12 h day/night cycle. Data presented in Figure 1, Figure 2 and Figure 3 were obtained from plants at this specific growth stage. Scale bar within the picture is 2 cm. (**B**) Phenotype of the wildtype (Col-0 and Ler) and two independent *HPR1* mutant alleles (*hpr1-1* and *hpr1-2*; [33]) grown in normal air (390 ppm CO_2_) for ~7 weeks in a 12/12 h day/night cycle. The photograph illustrates that at this stage the growth retardation of *hpr1* mutants is minor and onset of flowering similar between the mutants and the wildtype. Therefore, the metabolome analysis during development is comparable between both genotypes. (**C**) Phenotype at growth stage 5.1 [41] in short day (upper panel, after ~7 weeks) and long day (lower panel, ~4 weeks), at which leaf-material for metabolome analysis presented in Figure 6 was taken in both photoperiods. Scale bar within the picture is 2 cm.

Taking advantage of a ^14^C-glycine labeling approach, a more global overview on the carbon allocation in *hpr1* (Figure 2A) was obtained. Compared to the wild type, a larger fraction of photo-assimilated carbon was shifted towards amino acids biosynthesis in *hpr1*. Significant reductions were seen in the fractions combining either, organic acids, soluble sugars and high molecular mass components including starch, the cell wall and proteins (Figure 2A). Rationally, the increase in the amino acid pool was presumably due to the accumulation of photorespiratory glycine and serine (Figure 1A,B, Figure 4; Figure 6), while *hpr1* also showed some accumulations in other amino acids (Figure 5). A part of the explanation for amino acid accumulation could also be enhanced chlorophyll and protein degradation due to the decreased carbohydrate availability. This hypothesis is, at least to some extent, supported by elevated urea and putrescine levels in *hpr1* (Figure 5B). Moreover, *hpr1* contains reduced chlorophyll amounts [34] and has yellowish leaves in air (Figure A2). Thus, it seems likely that proteins are used as alternative respiratory substrates under circumstances were carbon is limited as suggested [53]. Such metabolic reprograming agrees well also with the alteration in the TCA-cycle intermediates observed during this study (Figure 5A). 

The reduction in either, the fraction of soluble sugars (mainly glucose, sucrose and fructose), as well as higher molecular/storage carbohydrates including starch and intermediates related to cell wall biosynthesis (raffinose, galactinol, myo-inositol) was present on a short-term during the ^14^C-labeling approach and consistently seen during plant development in *hpr1* (Figure 2A, Figure 4A). While reduction in the soluble sugars and starch was seen for other photorespiratory mutants before, e.g., [7,13,23], mainly because of impaired Calvin–Benson cycle operation and carbon export thereof, the likely additional impact on cell wall biosynthesis was not yet reported. However, it is possible that the general reduction in the availability of soluble sugars impacts on the synthesis of higher molecular mass carbohydrates. This effect seems to extent to a decrease in cell wall components (Figure 2A, Figure 4B). In addition to the lower substrate availability, a strong decrease in the transcript abundance of the major *myo*-inositol biosynthesizing enzyme *myo*-inositol 1-phosphate synthase 1 was observed (MIPS1, [40]) in *hpr1* during CO_2_ transition (Figure A1). Compared to that genes encoding galactinol, raffinose and stachyose synthases were not differentially expressed in the same experiment [7]. Collectively, it is likely to hypothesize that a combination of lowered substrate availability and deregulated MIPS1 account for the reduction in higher molecular mass carbohydrates including cell wall constituents. This altered metabolism might also serve as an explanation for the thinner and softer *hpr1* leaves (Figure A2) [33].

### 3.3. HPR1 Deficiency Impacts on Glutathione Biosynthesis

During photorespiration, HPR1 converts 3HP to glycerate in peroxisomes, using NADH as cofactor [34]. Therefore, it was tempting to speculate that lack of HPR1 eventually impacts on the peroxisomal redox-homeostasis and, in turn, the overall cellular redox status through the action of the malate valve [54]. To encounter imbalances in the subcellular redox-status, and to ultimately prevent oxidative damage, plants synthesize and accumulate glutathione (GSH) as redox-buffer in various subcellular compartments [55]. Indeed, we measured a significant increase (2-fold) in total GSH in *hpr1* during illumination (Figure 3A), suggesting alterations in the subcellular redox-state. Given we did not further characterize redox-metabolism in *hpr1* during this study, future work is needed in order to shed more light on this aspect. However, impairment of the photorespiratory flux in *hpr1* not only leads to the specific accumulation of GSH, but also to that of *O*-acetylserine (OAS) and cysteine compared to the wild type (Figure 3A). Therefore, and in light of the absolute increases in glycine and serine (Figure 1; Figure 6), the changes in GSH are more likely due to a partially redirected carbon flux from both amino acids towards GSH in illuminated leaves as observed for ETA via SDC (Figure 1). We can currently only hypothesize that increased cellular GSH is to protect cellular membranes are a matter of a general stress response as previously discussed [56].

### 3.4. Impairment of the Photorespiratory Flux in hpr1 Is Strongest during Vegetative Growth in a Fully Developed Rosette

Photorespiration is essential for the transition from heterotrophic to photoautotrophic growth, i.e., to establish photosynthesis in air [36,37]. Therefore, one can assume similar rates of photorespiration during plant development including equal abundances of photorespiratory intermediates. Indeed, the wild type showed only very minor alterations in the photorespiratory intermediates during both, vegetative and regenerative growth, respectively (Figure 4A). However, a slowdown of the photorespiratory flux in *hpr1* leads to a specific accumulation pattern of all pathway intermediates, that is increasing accumulation until onset of illumination, followed by decreasing amounts during regenerative growth (Figure 4A). From this result, it is reasonable to conclude that the need for a high photorespiratory flux is more required in the developing rosette but declines once plant shifts its metabolism to the reproductive phase. Accordingly, the growth reduction of *hpr1* was more pronounced during the vegetative phase, while the mutant is of similar size in the reproductive growth phase (Figure A2) [33,34].

### 3.5. A Shorter Photoperiod Intensifies the Photorespiratory Defect, Mainly at the GDC Step

Photoperiods can differentially affect plant metabolism including photorespiration. For example, by using Arabidopsis mutants deficient in peroxisomal catalase (*cat2*), it was shown that a longer photoperiod (16/8 h day/night cycle) increases phenotypic symptoms (i.e., formation of leaf-lesions) related to H_2_O_2_ accumulation, while a shorter photoperiod (8/16 h day/night cycle) enhances the perturbation of the intracellular redox state [42,43]. Considering the latter fact in combination with the presented day/night metabolite data of the wild type and the *hpr1* mutant grown in short- and long-days (Figure 6A,B), it seems reasonable to conclude that such shifts in the cellular redox state in shorter photoperiods mainly affect the activities of glycine decarboxylase (GDC) and, perhaps, glycerate kinase (GLYK) during the daily diurnal cycle. This statement is in good agreement with the observation that the photorespiratory intermediates glycine and glycerate showed the highest accumulation amplitude after onset of illumination in short days. Compared to that, glycolate, serine and 3HP showed only minor fluctuations that are also largely invariant between the two photoperiods in the wild type (Figure 6A). However, the described effects are significantly amplified in *hpr1*, displaying an impaired redox status (Figure 3A), especially at the glycine-to-serine conversion catalyzed by GDC (Figure 6B).

Onset of illumination induces photorespiration, which ultimately leads to a massive production of NADH in mitochondria through GDC activity [22]. Interestingly, it is known that GDC becomes inhibited at high NADH/NAD^+^ ratios [57], which eventually represents a mechanism to protect the mitochondria from overreduction if NADH recycling is suboptimal [14]. The strongly amplified glycine accumulation in the *hpr1* mutant, which is unable to directly re-oxidize NADH in peroxisomes, supports this statement. It seems likely that wild-type mitochondria are susceptible towards this mechanism to control GDC activity especially under short-days, given that glycine accumulates to a much lesser extent under long-days in the wild type and *hpr1* (Figure 6A,B). Accordingly, growth retardation of *hpr1* compared to the wildtype is more pronounced under short-days (Figure A2). Finally, this hypothesis could potentially also explain, why overexpression of the GDC H- and L-protein stimulates photosynthesis and plant growth mainly under shorter photoperiods [15,16].

Redox regulation of GLYK was observed only for C4 plants to date [58]. Therefore, the higher amplitude of glycerate accumulation in short-days might not be a matter of impaired redox regulation of the GLYK protein, but rather be explained better by relatively low light-induction of the GLYK protein amounts during the day/night cycle compared to other photorespiratory proteins [35]. Another argument against redox regulation of the C3 plant GLYK protein is the fact that glycerate fluctuations are comparable between the wild type and *hpr1* in short-days (Figure 6). However, elevation of glycerate amounts in *hpr1* grown under long-days are likely due to the higher fraction of 3HP conversion in the cytosol and the need to reincorporate the resulting glycerate into the chloroplast, where the reimport eventually limits the overall photorespiratory flux.

### 3.6. Conclusion

We have shown that primary metabolism in an Arabidopsis mutant deficient in *HPR1* is capable to adapt to moderate impairments in the photorespiratory flux to allow proper plant growth in normal air. In addition to a functional cytosolic HPR2 bypass [33], this is achieved via usage of compensatory mechanisms, preventing overaccumulation of inhibitory pathway intermediates. This includes non-enzymatic decarboxylation of 3HP and alternative serine degrading routes, including increased flux towards ETA and GSH (Figure 1 and Figure 3). Draining carbon from the core photorespiratory cycle towards other metabolisms, however, seems to reduce the overall leaf-carbon status, ultimately reducing starch and cell wall biosynthesis (Figure 4 and Figure 5, Figure A1). These reductions cause phenotypic alterations, particularly in short-days and during vegetative growth, eventually resulting from impaired GDC activity due to mitochondrial redox imbalances. This negative feed-back becomes alleviated in log-days and the transition to regenerative growth, where *hpr1* is of similar size as the wildtype (Figure A2). Collectively, the moderate phenotype of *hpr1*, lacking strong pleiotropic responses, will be a valuable tool in the future in order to identify the underlying regulation mechanisms of photorespiration and other cellular metabolic routes.

## 4. Materials and Methods

### 4.1. Plant Material and Growth

During this study *Arabidopsis thaliana* (Arabidopsis) ecotype Columbia (Col-0) was used as wild-type reference. A T-DNA insertional line of hydroxypyruvate reductase 1 (HPR1, *hpr1-1*, SALK067724) was obtained from the Nottingham Arabidopsis Stock Centre and homozygous plants isolated as previously described [33]. Seeds were sterilized with chloric acid, sown on soil and incubated at 4 °C to break dormancy for at least 2 days. Plants were grown in controlled environment chambers (Percival or Conviron; 12/12 h day/night cycle, 22/18 °C, ~120 µmol·m^−2^·s^−1^ irradiance, 390 µL L^−1^ CO_2_) on a 4:1 mixture of soil (Type Mini Tray; Einheitserdewerk, Uetersen, Germany) and vermiculite and regularly watered with 0.2% Wuxal liquid fertilizer (Aglukon, Düsseldorf, Germany). Where stated in the text, the photoperiod was changed to a 10/14 h (short-day) or 16/8 h (long-day) day/night cycle, with otherwise equal conditions. For most experiments we used plants at growth stage 5.1 [41].

### 4.2. Metabolite Analysis

#### 4.2.1. Gas-Chromatography Coupled to Mass Spectrometry (GC-MS)

To determine relative metabolite abundances during the development of Arabidopsis rosettes, the wildtype and the *hpr1* mutant were grown in normal air with a balanced photoperiod (12/12 h day/night cycle). Approximately 50 mg of leaf-material from five different biological individuals was harvested in the middle of the light phase (6 h illumination) after 3, 4, 5, 6 and 7 weeks after germination (WAG). For the analysis of metabolic alterations during growth in different photoperiods, we grew the wildtype and *hpr1* in normal air and harvested ~50 mg leaf-material from five biological replicates (growth stage 5.1. [41]) at four different time points during a day/night cycle as follows: short-day (10/14 h day/night cycle)—mid of day (MoD, 5 h illumination), end of day (EoD, 9 h illumination), mid of night (MoN, 7 h darkness) and end of night (EoN, 13 h darkness) and long-day (16/8 h day/night cycle)—MoD (8 h illumination), EoD (15 h illumination), MoN (4 h darkness) and EoN (7 h darkness). At each time point leaf material was harvested, immediately frozen in liquid nitrogen and stored at −80 °C until further processing as recommended before [59]. Metabolite extraction, derivatization, and analysis were performed as described previously [60,61]. Whereas stated in the text that values were determined as absolute metabolite contents, calibration curves of the respective metabolites were included in the same batch.

#### 4.2.2. High-Performance Liquid Chromatography (HPLC)

Quantification of the cysteine precursor *O*-acetylserine (OAS), the thiols (cysteine and glutathione—GSH+GSSG) and adenosine phosphates (ADP, ATP) was performed after selective labeling with fluorescent dyes and separation of the resulting metabolite-conjugates by HPLC as described previously [62,63]. For this purpose, we used ~100 mg of leaf tissue harvested from the wildtype and the *hpr1* mutant grown in normal air (12/12 h day/night cycle, EoD—9 h illumination) to growth stage 5.1 [41].

### 4.3. ^14^C-Glycine and ^13^C-Glucose-Feeding and Determination of Isotope Accumulation

For this purpose, we grew the wild type and the *hpr1-1* mutant in normal air (12/12 h day/night cycle) to growth stage 5.1 [41]. For the [U-^14^C]-glycine feeding experiment fully expanded leaves from at least five biological replicates were harvested from five-weeks-old plants after 4 h of illumination. Five leaf-discs (6 mm diameter) were cut from the leaf underwater and incubated in 5 mL of 5 mM HEPES-KOH, pH 7.0, containing 10 mM glycine supplemented with 37 kBq of [U-^14^C]-glycine (final specific radioactivity of 740 kBq mmol^−1^). The leaf-discs were incubated for 6 h under plant growth conditions (22 °C, light—120 µmol m^−2^ s^−1^, 390 ppm CO_2_ and 21% O_2_, and 70% relative humidity). Afterwards, samples were washed three times in unlabeled incubation medium, frozen in liquid nitrogen and stored at –80 °C until further analysis, as described by Fernie et al. [64]. During the incubation period the ^14^CO_2_ evolved was captured (in hourly intervals) in a KOH trap and the amount of released radiolabel was subsequently quantified by liquid scintillation counting.

For ^13^C-glucose feeding, we prepared 5 leaf-discs from at least 5 biological individuals after 4 h of illumination during the day/night cycle and incubated them for 2 h in a 10 mM MES-KOH solution (pH 6.5), containing either 10 mM [U-^12^C]-glucose or [U-^13^C]-glucose for 2 h (in principle MoD, 6 h after onset of illumination) under the same plant growth conditions mentioned above. The leaf discs were harvested after three washing steps with 10 mM MES-KOH solution (pH 6.5) by freezing with liquid nitrogen and stored at −80 until further analysis. Metabolite extraction, quantification and determination of the molecular accumulation of isotope was carried out as described before [60,65].

### 4.4. Statistical Analysis

As values are described to be significantly different from the control within the text, the differences were determined due to the performance of the two tailed Student’s *t*-test algorithm incorporated into Microsoft Excel 10.0 (Microsoft, Seattle, WA, USA).

## Data Availability

The data presented in this study are available in article.
